# Boosting the Self‐Trapped Exciton Emission in Alloyed Cs_2_(Ag/Na)InCl_6_ Double Perovskite via Cu^+^ Doping

**DOI:** 10.1002/advs.202103724

**Published:** 2022-01-17

**Authors:** Xingwen Cheng, Zhi Xie, Wei Zheng, Renfu Li, Zhonghua Deng, Datao Tu, Xiaoying Shang, Jin Xu, Zhongliang Gong, Xingjun Li, Xueyuan Chen

**Affiliations:** ^1^ CAS Key Laboratory of Design and Assembly of Functional Nanostructures Fujian Key Laboratory of Nanomaterials and State Key Laboratory of Structural Chemistry Fujian Institute of Research on the Structure of Matter Chinese Academy of Sciences Fuzhou Fujian 350002 China; ^2^ College of Mechanical and Electronic Engineering Fujian Agriculture and Forestry University Fuzhou Fujian 350002 China; ^3^ Fujian Science & Technology Innovation Laboratory for Optoelectronic Information of China Fuzhou Fujian 350108 China

**Keywords:** Cu^+^ doping, double perovskite, excited‐state dynamics, photoluminescence, self‐trapped exciton

## Abstract

Fundamental understanding of the effect of doping on the optical properties of 3D double perovskites (DPs) especially the dynamics of self‐trapped excitons (STEs) is of vital importance for their optoelectronic applications. Herein, a unique strategy via Cu^+^ doping to achieve efficient STE emission in the alloyed lead‐free Cs_2_(Ag/Na)InCl_6_ DPs is reported. A small amount (1.0 mol%) of Cu^+^ doping results in boosted STE emission in the crystals, with photoluminescence (PL) quantum yield increasing from 19.0% to 62.6% and excitation band shifting from 310 to 365 nm. Temperature‐dependent PL and femtosecond transient absorption spectroscopies reveal that the remarkable PL enhancement originates from the increased radiative recombination rate and density of STEs, as a result of symmetry breakdown of the STE wavefunction at the octahedral Ag^+^ site. These findings provide deep insights into the STE dynamics in Cu^+^‐doped Cs_2_(Ag/Na)InCl_6_, thereby laying a foundation for the future design of new lead‐free DPs with efficient STE emission.

## Graphical abstract

A strategy of Cu^+^ doping is proposed to boost the self‐trapped exciton (STE) emission in alloyed Cs_2_(Ag/Na)InCl_6_ double perovskites, with photoluminescence (PL) quantum yield increasing from 19.0% to 62.6% and excitation band shifting from 310 to 365 nm. Mechanistic investigation reveals that the remarkable PL enhancement stems from the increased radiative recombination rate and density of STEs.

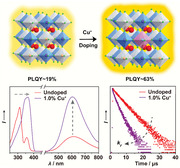

## 1 Introduction

All‐inorganic 3D lead‐free double perovskites (DPs) with the general formula A_2_B^I^B^III^X_6_ (A = Rb^+^, Cs^+^; B^I^ = K^+^, Ag^+^, Na^+^; B^III^ = In^3+^, Bi^3+^, Sb^3+^; X = Cl^−^, Br^−^, I^−^) have recently evoked considerable interest in a wide array of research fields owing to their intriguing optoelectronic properties.^[^ ^1^ ^]^ These DPs, while preserving the merits of 3D lead halide perovskites (typically CsPbX_3_) including long carrier diffusion distance, large absorption coefficient, and tunable bandgap, are superior to CsPbX_3_ in terms of the toxicity and stability issues, and the freedom of tunability via B site engineering.^[^ ^2^ ^]^ Therefore, they are ideal candidates as alternatives to lead halide perovskites and as a new generation of optoelectronic materials for various applications such as solar cells, photodetectors, and light‐emitting diodes (LEDs).^[^ ^3^ ^]^ In contrast to CsPbX_3_ with corner‐sharing [PbX_6_]^4−^ octahedra, the structural units of DPs are characterized by alternating [B^I^X_6_]^5−^ and [B^III^X_6_]^3−^ octahedra.^[^ ^4^ ^]^ As a result, the electronic dimensionality of DPs is reduced in comparison with that of CsPbX_3_, leading to many fascinating characteristics such as strong electron–phonon coupling, large exciton binding energy, and broadband self‐trapped exciton (STE) emission with a large Stokes shift.^[^ ^5^ ^]^ Nonetheless, because of the nature of indirect bandgap or direct bandgap with parity‐forbidden transitions, these DPs normally suffer from a low photoluminescence (PL) efficiency,^[^ ^6^ ^]^ which limits their application as light‐emitting materials in many technological fields.

To circumvent the limitation of DPs, it is essential to modify their bandgap and transition attributes by engineering the B site of the materials through metal ion doping or alloying.^[^ ^7^ ^]^ In this regard, d‐, f‐, and s‐electron ions such as Mn^2+^, Cr^3+^, Tb^3+^, Yb^3+^, Er^3+^, Bi^3+^, In^3+^, and Sb^3+^ have been established as effective dopants in DPs to modulate their electronic structures and boost their PL efficiencies.^[^ ^8^ ^]^ Specifically, broadband white‐light emission with a quantum yield (QY) of 86% was realized in the alloyed Cs_2_(Ag_0.60_/Na_0.40_)InCl_6_: Bi^3+^ crystals by breaking the parity‐forbidden transition through lowering the symmetry of the STE wavefunction.^[^ ^3a^ ^]^ However, the maximum excitation band of the crystals at the high energy region (325 nm) is not optimal for a commercial near‐UV (NUV, >350 nm) LED chip. Although many efforts have been devoted to tailoring both the excitation and emission of STEs in Cs_2_(Ag/Na)InCl_6_ DPs,^[^ ^9^ ^]^ the fundamental photophysics involved in the doping effect especially the dynamics of STEs remains elusive and not fully understood.

Herein, we report a unique strategy based on Cu^+^ doping to boost the STE emission in the alloyed Cs_2_(Ag/Na)InCl_6_ DPs. The effect of Cu^+^ doping on the electronic structure and optical properties of Cs_2_(Ag/Na)InCl_6_ and the STE dynamics are comprehensively surveyed by means of temperature‐dependent PL and ultrafast femtosecond transient absorption (fs‐TA) spectroscopies. The as‐synthesized Cs_2_(Ag/Na)InCl_6_: Cu^+^ crystals exhibit significantly enhanced PL stemming from the increased radiative recombination rate of STEs as well as the improved STE density. Furthermore, we demonstrate the excellent air, structural, and thermal stability of these Cu^+^‐doped Cs_2_(Ag/Na)InCl_6_ crystals and reveal their great potentials as efficient yellow‐emitting phosphors for application in NUV‐converted WLEDs.

## 2 Results and Discussion

**Figure 1:**
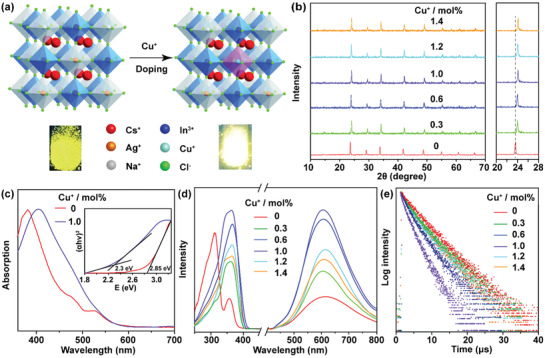
a) Crystal structure of Cs_2_(Ag/Na)InCl_6_ and the crystallographic site for Cu^+^ dopants. The PL photographs (*λ*_ex_ = 365 nm) for Cs_2_(Ag/Na)InCl_6_ and Cs_2_(Ag/Na)InCl_6_: 1.0%Cu^+^ powders are presented, showing significantly enhanced PL of the crystals upon Cu^+^ doping. b) Powder XRD patterns of Cs_2_(Ag/Na)InCl_6_: *x*%Cu^+^ with different Cu^+^ doping concentrations. The enlarged 2*θ* range (20–28°) of XRD patterns shows a monotonic shift of the diffraction peaks to higher angle with increasing the Cu^+^ concentration. c) Optical absorption spectra of Cs_2_(Ag/Na)InCl_6_ and Cs_2_(Ag/Na)InCl_6_: 1.0%Cu^+^. The inset shows the corresponding Tauc plots of the absorption spectra. d) PL excitation spectra (*λ*_em_ = 605 nm), PL emission spectra (*λ*_ex_ = 365 nm), and e) PL decay curves (*λ*_em_ = 605 nm) of Cs_2_(Ag/Na)InCl_6_: *x*%Cu^+^ with different Cu^+^ doping concentrations.

The alloyed Cs_2_(Ag/Na)InCl_6_ crystal is characterized by a 3D DP structure (space group *Fm‾3m*) consisting of alternating [MCl_6_]^5−^ (M = Ag or Na) and [InCl_6_]^3−^ corner‐sharing octahedra with Cs^+^ ions occupying the voids in between (**Figure** **1**a).^[^ ^4^ ^]^ Cu^+^ dopants are supposed to substitute the octahedral Ag^+^ site.^[^ ^10^ ^]^ Cs_2_(Ag/Na)InCl_6_: *x*%Cu^+^ single crystals with different Na–Ag alloying ratios and Cu^+^ doping concentrations were synthesized via a modified solvothermal method. The as‐synthesized crystals have a mean size around 100–400 µm (Figure S1, Supporting Information). The Na–Ag alloying ratios and Cu^+^ doping concentrations were controlled by varying the feeding ratios of the metal precursors between Na, Ag, and Cu, and checked by inductively coupled plasma–atomic emission spectroscopy (ICP–AES). The results show that the actual Na–Ag alloying ratios of the crystals were generally consistent with its feeding ratios (Table S1, Supporting Information), and an optimal Na–Ag alloying ratio of 2:3 was identified by powder X‐ray diffraction (XRD) and PL measurements (Figures S2 and S3, Supporting Information). To this regard, we fixed the Na–Ag alloying ratio of 2:3 and investigated the effect of Cu^+^ doping on the optical properties of the alloyed Cs_2_(Ag/Na)InCl_6_ (the formula was used throughout for convenience). In contrast to that of Na–Ag alloying, only a small amount of Cu^+^ (1.4 mol%) was found in the crystals even at a high feeding ratio (40 mol%) of Cu to (Na + Ag) (Table S2, Supporting Information). Powder XRD measurements show that all diffraction peaks of the Cu^+^‐doped samples can be well indexed into cubic Cs_2_AgInCl_6_ (ICSD No. 19 130) without any observable impurities, indicating pure phase and high crystallinity of the resulting crystals (Figure 1b). The diffraction peaks of the crystals (e.g., at 23.6°) shift to higher angles with increasing the Cu^+^ concentration, suggesting a lattice contraction induced by the substitution of Ag^+^ (0.126 nm) with smaller Cu^+^ (0.096 nm). X‐ray photoelectron spectroscopy (XPS) exhibits the signals of binding energies typical for Cs^+^ (3d), Ag^+^ (3d), Na^+^ (1s), In^3+^ (3d), Cl^–^ (2p), and Cu^+^ (2p) in Cs_2_(Ag/Na)InCl_6_: 1.0 mol%Cu^+^ (Figure S4, Supporting Information), confirming the monovalent state of Cu in Cs_2_(Ag/Na)InCl_6_ and its successful doping into the crystal lattice.

Figure 1c and Figure S5 (Supporting Information) show the optical absorption spectra of the undoped and Cu^+^‐doped Cs_2_(Ag/Na)InCl_6_ crystals. The crystals display a broad absorption band ranging from 350 to 650 nm, with its maximum shifting from 380 to 410 nm upon Cu^+^ doping. This indicates that Cu^+^ doping may lower the bandgap energy of Cs_2_(Ag/Na)InCl_6_ crystals, as confirmed by Tauc plots of the absorption spectra (inset of Figure 1c), whereby the optical bandgap of the crystals was estimated to decrease from 2.85 eV in Cs_2_(Ag/Na)InCl_6_ to 2.30 eV in Cs_2_(Ag/Na)InCl_6_: 1.0%Cu^+^. Such a bandgap decrease is related to the overlap of Cu‐3d orbital with Cl‐3p orbital in the valence band of the crystals,^[^ ^11^ ^]^ as confirmed by density‐functional theory (DFT) calculations. We chose a 5 × 1 × 1 supercell of 200 atoms to build the alloyed Cs_2_(Ag/Na)InCl_6_ system with 12 Ag atoms and 8 Na atoms, then one Cu atom was introduced to substitute one Ag atom, achieving the Cu^+^ doping concentration of 5.0 mol%. According to the calculated density of states (DOS) and band structures (Figure S6, Supporting Information), the conduction band minimum (CBM) of Cs_2_(Ag/Na)InCl_6_ is contributed mainly from In‐5s orbitals and the valence band maximum (VBM) is composed of Cl‐3p and Ag‐4d orbitals, resulting in a calculated bandgap of 1.37 eV. Upon Cu^+^ doping, the extra Cu‐3d orbitals overlap with a little Cl‐3p orbitals and form new bands located at 0.3–0.6 eV above the original VBM, and these new bands constitute new VBM for Cu^+^‐doped Cs_2_(Ag/Na)InCl_6_ system. As a result, the calculated bandgap is reduced to 0.68 eV in Cs_2_(Ag/Na)InCl_6_: 5%Cu^+^ system with a slight downshift (0.09 eV) of CBM. The calculated bandgaps are lower than the experimental values due to the general limitation of the generalized gradient approximation (GGA) scheme in DFT calculation.^[^ ^2^, ^8^, ^12^ ^]^ Despite of the underestimation of the calculated bandgaps and the higher Cu^+^ doping concentration (5.0 mol%) used for calculation, it can be concluded from the DFT calculation that Cu^+^ doping brings about a new VBM above the original VBM, which results in an evident bandgap reduction of Cs_2_(Ag/Na)InCl_6_ upon Cu^+^ doping.

Under UV excitation at 365 nm, the crystals exhibited bright yellow PL with a broad emission band (full‐width at half maximum (FWHM) of ≈590 meV) covering from 400 to 800 nm (Figure 1d), which can be ascribed to the intrinsic STE emission arising from the strong Jahn–Teller distortion of the [AgCl_6_]^5−^ octahedra.^[^ ^6a^ ^]^ The PL intensity of the crystals increased gradually with increasing the Cu^+^ doping concentration from 0 to 1.0 mol% and then decreased at higher Cu^+^ concentrations, in parallel with the increased PLQY of the crystals from 19.0% ± 0.4% in Cs_2_(Ag/Na)InCl_6_ to 62.6% ± 0.6% in Cs_2_(Ag/Na)InCl_6_: 1.0%Cu^+^. PL excitation spectra of the crystals showed an evident redshift in the optimal excitation band from 310 to 365 nm upon Cu^+^ doping (Figure 1d), which is highly desirable for their application as emitters in NUV‐converted WLEDs. Further wavelength‐dependent PL excitation and emission spectra showed identical excitation and emission bands (Figure S7, Supporting Information), confirming the single luminescent center of STEs in Cs_2_(Ag/Na)InCl_6_: 1.0%Cu^+^ crystals. Intriguingly, we observed that the enhancement in PL intensity and PLQY of the crystals was accompanied by a decrease in PL lifetime (Figure 1e; Figure S8, Supporting Information). The effective PL lifetime of the crystals decreased from 6.4 to 3.9 µs with increasing the Cu^+^ concentration from 0 to 1.0 mol% and then increased to 5.2 µs at 1.4 mol% of Cu^+^ doping (Table S3, Supporting Information). Such a change in PL intensity and PL lifetime of Cs_2_(Ag/Na)InCl_6_: *x*%Cu^+^ with varying Cu^+^ concentrations is unusual and drastically different from the common observation that the PLQY improvement of a phosphor is concomitant with the increase in PL lifetime due to the suppression of nonradiative relaxation.^[^ ^13^ ^]^ It is known that the PLQY (Φ) of a phosphor is determined by the radiative (*k*_r_) and nonradiative (*k*_nr_) decay rates of its excited state and can be expressed as follows:^[^ ^14^ ^]^

(1) $$\Phi =\frac{k_{r}}{k_{r}+k_{nr}}\;=\frac{k_{r}}{k_{obs}}\;=\frac{\tau_{obs}}{\tau_{r}}\;$$

where *τ*_obs_ and *τ*_r_ represent the observed and radiative decay times, respectively. Therefore, we deduced that the improved PLQY along with the decreased PL lifetime observed in Cu^+^‐doped Cs_2_(Ag/Na)InCl_6_ originated mainly from the increased radiative decay (or recombination) rate of STEs caused by Cu^+^ doping. By taking the measured PL lifetimes into Equation (1), the radiative decay rate of STEs was derived, with the value increasing from 3.0 × 10^4^ s^−1^ in Cs_2_(Ag/Na)InCl_6_ to 1.6 × 10^5^ s^−1^ in Cs_2_(Ag/Na)InCl_6_: 1.0%Cu^+^ (Table S3, Supporting Information). The remarkable increase in radiative recombination rate of STEs can be attributed to the symmetry breakdown of the [AgCl_6_]^5−^ octahedron as well as the electron wavefunction at the Ag^+^ site due to Cu^+^ substitution‐induced lattice contraction, which may lead to a parity change in the STE wavefunction and thereby promote the radiative recombination rate of STEs, as previously reported by Tang et al. in Cs_2_(Ag/Na)InCl_6_: Bi^3+^ crystals.^[^ ^3a^ ^]^

**Figure 2:**
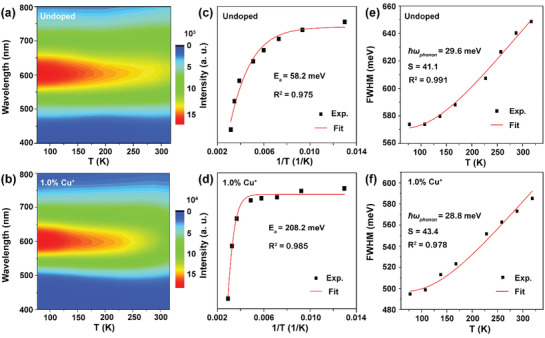
Contour plots of the temperature‐dependent PL emission spectra (*λ*_ex_ = 365 nm) of a) Cs_2_(Ag/Na)InCl_6_ and b) Cs_2_(Ag/Na)InCl_6_: 1.0%Cu^+^ in the temperature range of 77−320 K. Integrated PL intensity of STEs in c) Cs_2_(Ag/Na)InCl_6_ and d) Cs_2_(Ag/Na)InCl_6_: 1.0%Cu^+^ as a function of inverse temperature. FWHM of the STE emission in e) Cs_2_(Ag/Na)InCl_6_ and f) Cs_2_(Ag/Na)InCl_6_: 1.0%Cu^+^ as a function of temperature. The activation energy (*E*_a_), the Huang‐Rhys factor (*S*), and the phonon energy (*ћω*_phonon_) of the crystal lattice were derived by fitting to the data in (c)−(f).

To gain deep insights into the effect of Cu^+^ doping on the STE emission of Cs_2_(Ag/Na)InCl_6_, we carried out temperature‐dependent PL spectroscopic measurements. **Figure** **2**a,b and Figure S9 (Supporting Information) show the temperature‐dependent PL emission spectra (77−320 K) of the undoped and 1.0 mol% Cu^+^‐doped Cs_2_(Ag/Na)InCl_6_ crystals under excitation at 365 nm. The PL intensities of the crystals decreased gradually with increasing the temperature from 77 to 320 K, along with the emission bands broadening from 572 and 495 meV to 649 and 585 meV in Cs_2_(Ag/Na)InCl_6_ and Cs_2_(Ag/Na)InCl_6_: 1.0%Cu^+^, respectively, as a result of accelerated nonradiative relaxation and enhanced electron‐phonon coupling of STEs at higher temperatures.^[^ ^15^ ^]^ Based on the Arrhenius plot of the integrated PL intensity versus the inverse temperature (Figure 2c,d)^[^ ^16^ ^]^

(2) $$I\left(T\right)=\frac{I_{0}}{1+A\exp\left(-E_{a}/k_{b}T\right)}\;$$

where *I(T)* and *I*_0_ are integrated PL intensities at *T* and 0 K, respectively, A is a constant, *k*_b_ is the Boltzman constant, and *E*_a_ is the activation energy. The activation energies of STEs were determined to be 58.2 and 208.2 meV in Cs_2_(Ag/Na)InCl_6_ and Cs_2_(Ag/Na)InCl_6_: 1.0%Cu^+^, respectively. According to the Boltzmann distribution law and Fermi's Golden Rule, the radiative decay rate (*k*_r_) of excitons can be expressed as follows:^[^ ^17^ ^]^

(3) $$\begin{array}{ccc} k_{r}\left(T\right) & = & \frac{ne^{2}\omega^{2}}{3\varepsilon_{0}c^{3}m_{0}}\;f_{0}{\left(\frac{\mu }{M}E_{a}\right)}^{\frac{3}{2}}\;\frac{1-e^{-\Delta E/k_{b}T}}{\Delta E}\\ & = & C{\left(E_{a}\right)}^{\frac{3}{2}}\frac{1-e^{-\Delta E/k_{b}T}}{\Delta E} \end{array}$$

where C is a constant dictated by the refractive index (*n*), the transition frequency (*ω*), the vacuum permittivity (*ε_0_*), the electron mass (*m_0_*), the equivalent exciton mass (*µ*), and the exciton mass (*M*) of the materials, and *∆E* is the energy line width. It is clear that *k*_r_ is proportional to the 3/2 power of *E*_a_. Therefore, the radiative recombination rate (*k*_r_) of STEs in the crystals was significantly improved after Cu^+^ doping, in view of the larger activation energy (*E*_a_) of STEs in Cs_2_(Ag/Na)InCl_6_: 1.0%Cu^+^ (208.2 meV) than in Cs_2_(Ag/Na)InCl_6_ (58.2 meV). This agrees well with the observation that Cu^+^ doping resulted in decreased PL lifetime of the crystals. To unveil the influence of electron–phonon coupling, we analyzed the temperature dependence of the emission band broadening using the equation^[^ ^18^ ^]^

(4) $$FWHM\;=\;2.36\sqrt{S}\hbar \omega_{phonon}\sqrt{\coth\frac{\hbar \omega_{phonon}}{2k_{b}T}\;}$$

where *S* and *ћω*_phonon_ are Huang‐Rhys factor and phonon energy, respectively. The phonon energy (*ћω*_phonon_) and Huang‐Rhys factor (*S*) were calculated to be 29.6 meV and 41.1 for Cs_2_(Ag/Na)InCl_6_ and 28.8 meV and 43.4 for Cs_2_(Ag/Na)InCl_6_: 1.0%Cu^+^, respectively (Figure 2e,f), which are generally consistent with those reported in Cs_2_AgInCl_6_ and Cs_2_(Ag/Na)InCl_6_.^[^ ^3^, ^7^ ^]^ The fitted phonon energies were also close to that (36.5 meV) of the A_1g_ longitudinal optical phonon mode of [InCl_6_]^3−^ octahedron determined by Raman spectra (Figure S10, Supporting Information), suggesting a dominant phonon mode of A_1g_ involved in the electron−phonon coupling.^[^ ^19^ ^]^ Such large *S* values indicate strong electron−phonon coupling, which favors the formation of STEs in the soft lattice of Cs_2_(Ag/Na)InCl_6_: 1.0%Cu^+^ and accounts for the broadband (≈590 meV) and large Stokes shift (240 nm) of the STE emission (Figure S11, Supporting Information).

**Figure 3:**
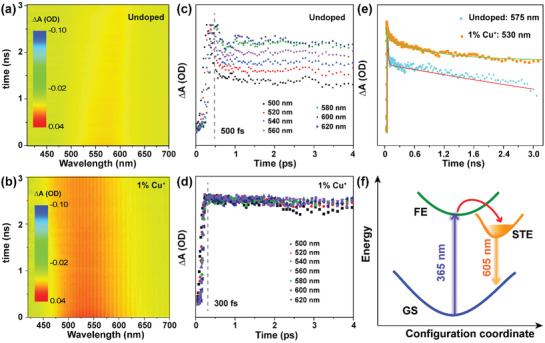
Contour plots of the fs‐TA spectra of a) Cs_2_(Ag/Na)InCl_6_ and b) Cs_2_(Ag/Na)InCl_6_: 1.0%Cu^+^ upon excitation with a 365 nm fs‐pulsed laser. The rise portion of the normalized PIA kinetic curves of c) Cs_2_(Ag/Na)InCl_6_ and d) Cs_2_(Ag/Na)InCl_6_: 1.0%Cu^+^ in the probe region from 500 to 620 nm. e) Normalized PIA decay curves of Cs_2_(Ag/Na)InCl_6_ at 575 nm and Cs_2_(Ag/Na)InCl_6_: 1.0%Cu^+^ at 530 nm. f) Configuration coordinate diagram of Cs_2_(Ag/Na)InCl_6_, showing the electronic transitions involved in the STE emission. GS and FE denote the ground state and free exciton, respectively.

To probe the ultrafast photophysical processes involved in the photoexcitation of the crystals, we performed fs‐TA spectroscopic measurements. **Figure** **3**a,b shows the contour plots of the fs‐TA spectra of the undoped and 1.0 mol% Cu^+^‐doped Cs_2_(Ag/Na)InCl_6_ upon excitation with a 365 nm fs‐pulsed laser under identical conditions. Both samples exhibited a positive photoinduced absorption (PIA) in the probe region from 470 to 640 nm, which can be an evidence for the formation of STEs in the crystal lattice.^[^ ^20^ ^]^ Moreover, it was observed that the PIA signal of Cu^+^‐doped sample was significantly enhanced and blue‐shifted to 530 nm in comparison with that (575 nm) of the undoped one. The blueshift in PIA of STEs is due to the effect of Cu^+^ alloying on the bandgap of Cs_2_(Ag/Na)InCl_6_, as proved by the steady‐state optical absorption spectra. The enhanced PIA of STEs implies that Cu^+^ doping may increase the density of the photo‐generated STEs in Cs_2_(Ag/Na)InCl_6_ lattice and thereby boost the STE emission. Further kinetic traces of PIA in the probe region from 500 to 620 nm displayed an identical rise time of ≈500 and ≈300 fs for the undoped and Cu^+^‐doped samples, respectively (Figure 3c,d), indicating that the broadband PIA of both samples originated from a single excited STE state. Such an ultrafast rise time in PIA is closely related to the Jahn–Teller distortion of [AgCl_6_]^5−^ octahedra, which agrees well with the STE formation time as previously reported by Han et al.^[^ ^20b^ ^]^ Therefore, the formation time of STEs in the crystals decreased after Cu^+^ doping, which is beneficial for STE generation and accounts for the increased STE density in Cu^+^‐doped sample. Figure 3e compares the PIA decay curves of Cs_2_(Ag/Na)InCl_6_ at 575 nm and Cs_2_(Ag/Na)InCl_6_: 1.0%Cu^+^ at 530 nm. By biexponential fitting to the PIA decay curves, two distinct decay components were derived: a fast decay associated with the nonradiative relaxation of STEs to the quenching centers such as lattice or surface defects, and a slow decay related to the radiative recombination of STEs through photon emission.^[^ ^9b^ ^]^ It was found that the contribution of nonradiative relaxation in Cs_2_(Ag/Na)InCl_6_: 1.0%Cu^+^ (23.7%) was much less than that in pristine Cs_2_(Ag/Na)InCl_6_ (48.2%; Table S4, Supporting Information). These results demonstrate unambiguously that Cu^+^ doping promotes the radiative recombination process of STEs in Cs_2_(Ag/Na)InCl_6_. Based on the above analyses, the dynamics of STEs in Cs_2_(Ag/Na)InCl_6_: Cu^+^ crystals was unveiled. As illustrated in Figure 3f, under NUV excitation at 365 nm, free excitons are created and then trapped by the distorted [AgCl_6_]^5−^ octahedra owing to the Jahn–Teller effect, forming STEs in the bandgap of the crystals.^[^ ^9b^ ^]^ The radiative recombination of STEs yields the broadband emission at ≈605 nm with a large Stokes shift. Upon Cu^+^ doping, the radiative recombination probability of STEs as well as the STE density is greatly improved due to the symmetry breakdown of the STE wavefunction, which leads to the boosted STE emission.

**Figure 4:**
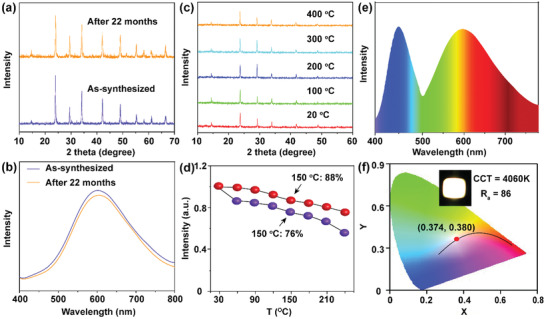
a) Powder XRD pattern and b) PL emission spectrum (*λ*_ex_ = 365 nm) of the as‐synthesized Cs_2_(Ag/Na)InCl_6_: 1.0%Cu^+^ crystals and the corresponding patterns after their exposure to ambient air for 22 months. c) Temperature‐dependent XRD patterns of Cs_2_(Ag/Na)InCl_6_: 1.0%Cu^+^ in the temperature range of 20–400 °C. d) Integrated PL intensities of Cs_2_(Ag/Na)InCl_6_ (violet ball) and Cs_2_(Ag/Na)InCl_6_: 1.0%Cu^+^ (red ball) as a function of temperature. e) EL spectrum of the NUV‐converted WLED based on the mixtures of yellow‐emitting Cs_2_(Ag/Na)InCl_6_: 1.0%Cu^+^ and blue‐emitting BaMgAl_10_O_7_:Eu^2+^ at a drive current of 25 mA. f) CIE coordinates of the NUV‐converted WLED. The inset shows the corresponding EL photograph of the WLED.

Furthermore, we evaluated the stability of Cs_2_(Ag/Na)InCl_6_: Cu^+^ crystals. The XRD pattern, PL spectrum, and PL intensity of Cs_2_(Ag/Na)InCl_6_: 1.0%Cu^+^ remained essentially unchanged after their storage in the air over 22 months, manifesting the excellent air stability of the crystals (**Figure** **4**a,b). Temperature‐dependent XRD measurements showed that the diffraction peaks of the crystals were nearly the same and matched well with cubic Cs_2_AgInCl_6_ (ICSD No. 19130) in the temperature range of 20–400 °C (Figure 4c), revealing the prominent structure stability of the crystals. Further temperature‐dependent PL emission spectra showed that Cu^+^ doping can also improve the thermal stability of Cs_2_(Ag/Na)InCl_6_ crystal (Figure S12, Supporting Information). The integrated PL intensities of the undoped and Cu^+^‐doped samples remained 76.2% and 88.4%, respectively, at 150 °C compared with their initial intensities at room temperature (RT; Figure 4d). Such outstanding air, structure, and thermal stability of Cs_2_(Ag/Na)InCl_6_: Cu^+^ crystals, along with the high PLQY (62.6%) and broad emission band at ≈605 nm, makes them an ideal candidate as an efficient yellow‐emitting phosphor in WLED. For this purpose, we fabricated a WLED by encapsulating Cs_2_(Ag/Na)InCl_6_: 1.0%Cu^+^ with the blue phosphor BaMgAl_10_O_7_:Eu^2+^ onto a commercial 365‐nm NUV chip. The as‐fabricated WLED device exhibited bright electroluminescence (EL) at a drive current of 25 mA, with a color coordinate of (0.374,0.380) in CIE 1931, a color‐rendering index (*R*_a_) of 86, and a white light correlated color temperature (CCT) of 4060 K (Figure 4e,f), thus validating the promise of Cs_2_(Ag/Na)InCl_6_: Cu^+^ DPs in solid‐state lighting.

## 3 Conclusions

In summary, we have systematically investigated the optical properties and excited‐state dynamics of Cu^+^‐doped Cs_2_(Ag/Na)InCl_6_ crystals. The substitution of Ag^+^ by a small amount (1.0 mol%) of Cu^+^ resulted in the boosted STE emission of Cs_2_(Ag/Na)InCl_6_ at 605 nm, with PLQY increasing from 19.0% to 62.6% and excitation band shifting from 310 to 365 nm. Mechanistic investigation through temperature‐dependent PL and femtosecond transient absorption spectroscopies unraveled that the remarkable PL enhancement was ascribed to the increased density and radiative recombination rate of STEs, as a result of symmetry breakdown of the STE wavefunction at the octahedral Ag^+^ site induced by Cu^+^ doping. Besides, Cu^+^ doping also improved the thermal stability of the crystals, retaining 88.4% of the RT PL intensity at 150 °C. These results reveal the great potential of Cu^+^‐doped Cs_2_(Ag/Na)InCl_6_ as an efficient NUV‐converted yellow‐emitting phosphor in WLEDs, which may open up a new avenue for the exploration of novel luminescent lead‐free DPs via metal ion doping toward versatile applications such as solid‐state lighting.

## 4 Experimental Section

### Chemicals and Materials

CsCl (99.9%), AgCl (99.9%), CuCl (99.9%), NaCl (99.9%), and isopropanol (99.5%) were purchased from Aladdin (Shanghai, China). In(CH_3_CO_2_)_3_ (99.99%) was purchased from Sigma–Aldrich (Shanghai, China). HCl was of analytical grade and purchased from Sinopharm Chemical Reagent Co. (Shanghai, China). All the chemical reagents were used as received without further purification.

### Synthesis of Cs_2_(Ag/Na)InCl_6_ Single Crystals

Cs_2_(Ag/Na)InCl_6_ single crystals were synthesized via a modified solvothermal method. In a typical synthesis of Cs_2_(Ag_0.6_/Na_0.4_)InCl_6_, 2 mmol of CsCl, 0.6 mmol of AgCl, 0.4 mmol of NaCl, and 1 mmol of In(CH_3_CO_2_)_3_ were mixed with 10 ml of HCl in a 25 ml Teflon‐lined autoclave. The mixture was heated to 180 °C for 12 h and then cooled down to RT naturally. The products were collected by centrifugation, washed twice with isopropanol, and finally dried at 60 °C in a vacuum for 24 h. For synthesizing Cs_2_(Ag/Na)InCl_6_ of different Na–Ag alloying ratios, different molar ratios of Na to Ag with 1 mol total amount of (Na + Ag) precursors were used under otherwise identical conditions. The actual Na–Ag alloying ratios of the crystals were identified by ICP–AES.

### Synthesis of Cu^+^‐Doped Cs_2_(Ag/Na)InCl_6_ Single Crystals

In a typical synthesis of 20 mol% (nominal concentration) Cu^+^‐doped Cs_2_(Ag/Na)InCl_6_, 2 mmol of CsCl, 0.6 mmol of AgCl, 0.4 mmol of NaCl, 0.2 mmol of CuCl, and 1 mmol of In(CH_3_CO_2_)_3_ were mixed with 10 ml of HCl in a 25 ml Teflon‐lined autoclave. The mixture was heated to 180 °C for 12 h and then cooled to RT naturally. The products were collected by centrifugation, washed twice with isopropanol, and finally dried at 60 °C in a vacuum for 24 h. For synthesizing Cs_2_(Ag/Na)InCl_6_: *x*%Cu^+^ of different Cu^+^ doping concentrations, different molar ratios of Cu to (Na + Ag) with fixed feeding amounts of Na (0.4 mmol of NaCl) and Ag (0.6 mmol of AgCl) were used under otherwise identical conditions. The nominal Cu^+^ doping concentration was defined by the molar ratio of Cu to (Na + Ag) in the precursors, and the actual Cu^+^ doping concentrations were identified by ICP–AES.

### Theoretical Calculations

DFT calculations were carried out using the Vienna ab initio simulation package (VASP). A 5 × 1 × 1 supercell of 200 atoms was chosen to build the alloyed Cs_2_(Ag/Na)InCl_6_ system, in which 8 of 20 Ag atoms were replaced by Na atoms, making Na–Ag ratio of 2:3. Furthermore, the Cu^+^‐doped Cs_2_(Ag/Na)InCl_6_ system was modeled with additional one Ag atom substituted by one Cu atom, creating Cu^+^ doping concentration of 5.0 mol%. The generalized gradient approximation (GGA) with Perdew–Burke–Ernzerhof (PBE) function was employed to describe exchange‐correlation interactions. The projector augmented wave (PAW) pseudopotentials were used to describe the electron–ion interactions. For expanding electronic wave functions, the cutoff energy of plane‐wave basis was set as 500 eV, and a Monkhorst–Pack k mesh of 1 × 5 × 5 was used to sample the Brillouin zone. The convergence threshold of 10^−6^ eV was adopted for total energy calculation, and the force convergence criteria were set as 0.02 eV Å^−1^ per atom in geometry relaxation.

### Structural and Optical Characterization

Powder XRD patterns were collected with an X‐ray diffractometer (MiniFlex2, Rigaku) using Cu K*α*1 radiation (*λ* = 0.154187 nm). ICP analysis was conducted on an ICP–AES spectrometer (Ultima2, Jobin Yvon). The scanning electron microscopy (SEM) measurements were performed by using a JSM‐6700F SEM. XPS measurements were performed on a Thermo Fisher ESCALAB 250Xi using Al K*α* (1486.6 eV) and He I*α* (220 eV) as the radiation sources. Raman spectra were recorded using a micro‐Raman spectrometer (Invia Reflex) with an excitation laser source of 532 nm. Optical absorption spectra were translated from the corresponding diffuse reflectance spectra that were recorded with a Perkin–Elmer Lambda 950 UV–vis–NIR spectrometer by using BaSO_4_ as a reference. PL excitation spectra, PL emission spectra, and PL decay curves were measured on the FLS980 spectrometer (Edinburgh) equipped with both continuous xenon (450 W) and pulsed flash lamps. PL photographs were taken by using a Huawei P30Pro cell phone without using any filter. The absolute PLQYs of the samples were measured by employing a standard barium sulfate coated integrating sphere (150 mm in diameter, Edinburgh) as the sample chamber that was mounted on the FLS980 spectrometer with the entry and output port of the sphere located in 90° geometry from each other. A standard tungsten lamp was used to correct the optical response of the instrument. For temperature‐dependent PL measurements, the samples were placed on the thermal stage (77–873 K, THMS 600, Linkam Scientific Instruments) and excited with the continuous xenon lamp. The ultrafast fs‐TA measurements were conducted under a pump wavelength of 365 nm on Helios (Ultrafast systems) spectrometers using a regeneratively amplified femtosecond Ti:sapphire laser system (Spitfire Pro‐F1KXP, Spectra‐Physics). All the spectral data were recorded at RT by using the powder samples unless otherwise noted and corrected for the spectral response of both the spectrometer and the integrating sphere.

## Conflict of Interest

The authors declare no conflict of interest.

## Supporting information

Supporting Information

## Data Availability

The data that support the findings of this study are available from the corresponding author upon reasonable request.
