# Supplementary material for: Boosting the Self‐Trapped Exciton Emission in Alloyed Cs2(Ag/Na)InCl6 Double Perovskite via Cu+ Doping
Source: Adv Sci (Weinh). 2022 Jan 17;9(7):2103724. doi: 10.1002/advs.202103724 (PMC8895137; doi:10.1002/advs.202103724)
Supplement: Supplementary file 1 — Supporting Information [file ADVS-9-2103724-s001.pdf]

ADVANCED SCIENCE
Open Access

Supporting Information

for *Adv. Sci.,* DOI: 10.1002/advs.202103724

Boosting the Self-Trapped Exciton Emission in Alloyed Cs2(Ag/Na)InCl6 Double Perovskite via Cu^{+} Doping

Xingwen Cheng, Zhi Xie, Wei Zheng, Renfu Li, Zhonghua Deng, Datao Tu, Xiaoying Shang, Jin Xu, Zhongliang Gong, Xingjun Li, and Xueyuan Chen**

Copyright WILEY-VCH Verlag GmbH & Co. KGaA, 69469 Weinheim, Germany, 2020.

Supporting Information

Boosting the Self-Trapped Exciton Emission in Alloyed $Cs_2(Ag/Na)InCl_6$ Double Perovskite via Cu^+ Doping

Xingwen Cheng[a], Zhi Xie[b], Wei Zheng[ac], Renfu Li[ac], Zhonghua Deng[a], Datao Tu[ac], Xiaoying Shang[a], Jin Xu[ac], Zhongliang Gong[a], Xingjun Li[ac], and Xueyuan Chen[ac]**

[a]CAS Key Laboratory of Design and Assembly of Functional Nanostructures, Fujian Key Laboratory of Nanomaterials, and State Key Laboratory of Structural Chemistry, Fujian Institute of Research on the Structure of Matter, Chinese Academy of Sciences, Fuzhou, Fujian 350002, China.

[b]College of Mechanical and Electronic Engineering, Fujian Agriculture and Forestry University, Fuzhou, Fujian 350002, China.

[c]Fujian Science & Technology Innovation Laboratory for Optoelectronic Information of China, Fuzhou, Fujian 350108, China.

E-mail: zhengwei@fjirsm.ac.cn; xchen@fjirsm.ac.cn

Table S1 Nominal and actual Na concentrations in $Cs_2(Ag/Na)InCl_6$ crystals synthesized at different feeding ratios of Na/Ag precursors. The nominal Na concentration was defined by the molar ratio of Na to (Na + Ag) in the precursors, and the actual Na concentrations were identified by inductively coupled plasma-atomic emission spectroscopy (ICP-AES).

Sample	Na : Ag	Nominal / mol% (Na)	Actual / mol% (Na)
1	0 : 1	0.0	0.0
2	3 : 17	15.0	14.2
3	3 : 7	30.0	28.4
4	2 : 3	40.0	39.1
5	3 : 2	60.0	59.6
6	4 : 1	80.0	78.3

Table S2 Nominal and actual Cu^+ doping concentrations in $Cs_2(Ag/Na)InCl_6$ crystals by varying the molar ratio of Cu to (Na + Ag) in the precursors. The nominal Cu^+ doping concentration was defined by the molar ratio of Cu to (Na + Ag) in the precursors, and the actual Cu^+ doping concentrations were identified by ICP-AES.

Sample	Cu : (Na + Ag)	Nominal / mol% (Cu^+)	Actual / mol% (Cu^+)
1	0 : 1	0.0	0
2	1 : 20	5.0	0.3
3	1 : 10	10.0	0.6
4	1 : 5	20.0	1.0
5	3 : 10	30.0	1.2
6	2 : 5	40.0	1.4

Table S3 Absolute photoluminescence (PL) quantum yield (QY), effective PL lifetime (τ_{eff}), radiative decay time (τ_r), and radiative decay rate (k_r) of $Cs_2(Ag/Na)InCl_6$: $x\%Cu^+$ with different Cu^+ doping concentrations upon ultraviolet (UV) excitation at 365 nm. τ_{eff}, τ_r and k_r were derived from the PL decay curves and PLQYs of the crystals. The absolute PLQY for each sample was measured independently for three times under identical conditions to yield the average value and standard deviation.

Cu^+ (%)	PLQY (%)	τ_{eff} / μs	τ_r / μs	k_r / s^{-1}
0	19.0 ± 0.4	6.4	33.7	3.0×10^4
0.3	40.5 ± 0.5	5.6	13.8	7.2×10^4
0.6	59.8 ± 0.5	4.5	7.5	1.3×10^5
1.0	62.6 ± 0.6	3.9	6.2	1.6×10^5
1.2	48.7 ± 0.4	5.3	10.9	9.2×10^4
1.4	45.2 ± 0.4	5.2	11.5	8.7×10^4

Table S4 Nonradiative relaxation time (τ_1), radiative relaxation time (τ_2) and their weight ratios (A_1, A_2) by biexponential fitting to the positive photoinduced absorption (PIA) decay curves of $Cs_2(Ag/Na)InCl_6$ and $Cs_2(Ag/Na)InCl_6$: 1.0%Cu^+.

Sample	τ_1 (ps)	A_1 (%)	τ_2 (ns)	A_2 (%)
Undoped	16.9	48.2	14.8	51.8
1.0% Cu^+	86.2	23.7	2.0	76.3

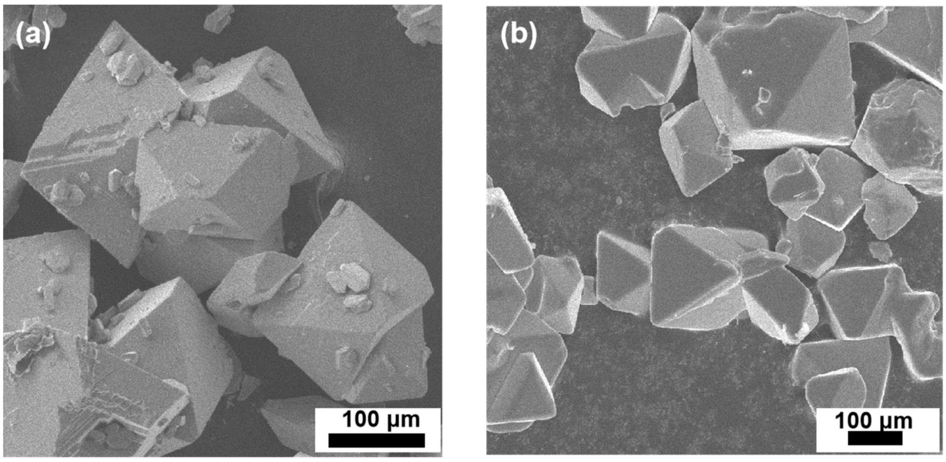

Figure S1 SEM images of the as-synthesized a) $Cs_2(Ag/Na)InCl_6$ and b) $Cs_2(Ag/Na)InCl_6$: 1.0% Cu^+ crystals, showing that the crystals have a mean size around 100–400 μm.

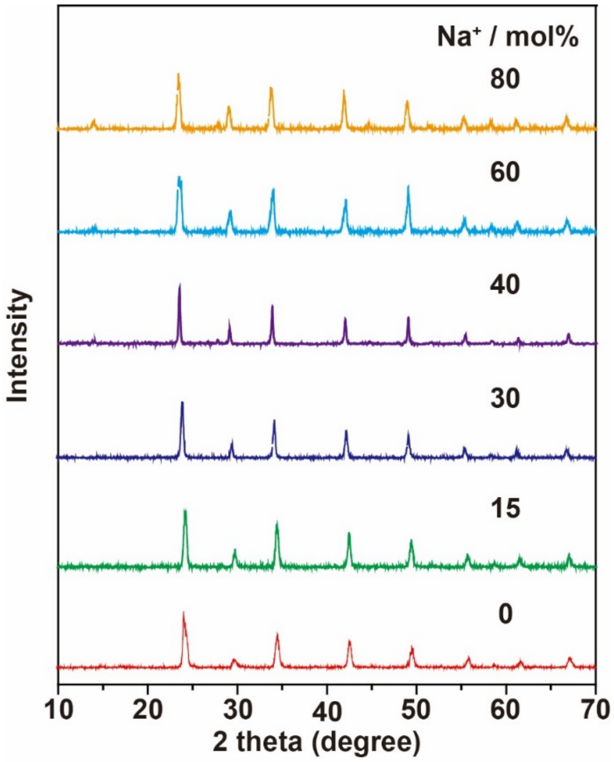

Figure S2 Powder XRD patterns of $Cs_2(Ag/Na)InCl_6$ crystals with different Na^+ concentrations. All the diffraction peaks of the crystals match well with those of cubic $Cs_2AgInCl_6$ (ICSD No. 19130), indicating high crystallinity and phase purity of the alloyed $Cs_2(Ag/Na)InCl_6$ crystals.

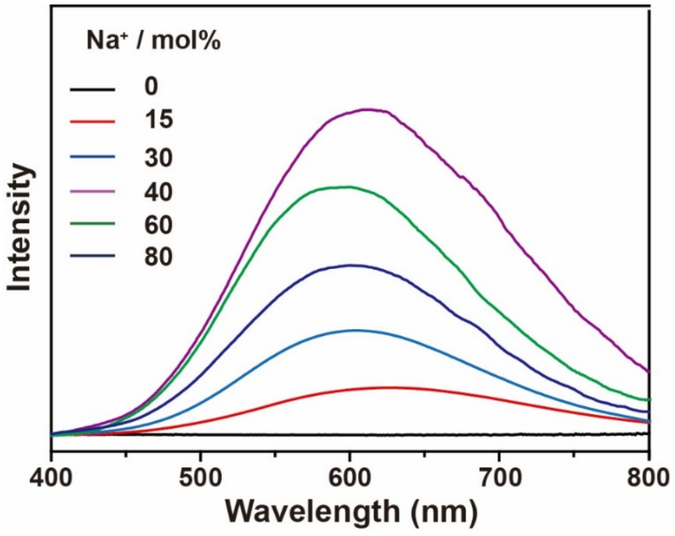

Figure S3 PL emission spectra (λ_{ex} = 365 nm) of $Cs_2(Ag/Na)InCl_6$ with different Na^+ concentrations. The optimal Na^+ concentration was determined to be 40 mol% in the crystals, which yields the most intense PL among the investigated Na^+ concentrations.

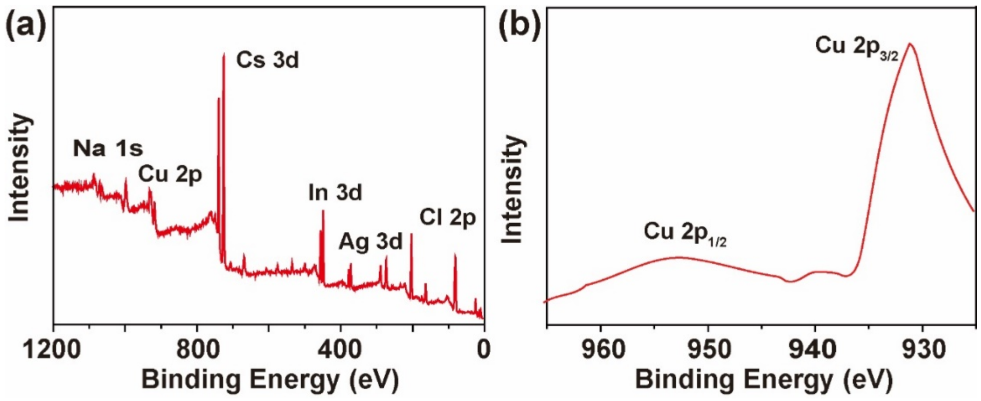

Figure S4 a) XPS spectrum and b) the corresponding high-resolution XPS spectrum of Cu^+ (2p) in $Cs_2(Ag/Na)InCl_6$: 1.0%Cu^+. The XPS spectrum is shown over the energy region typical for Cs^+ (3d), Ag^+ (3d), Na^+ (1s), In^{3+} (3d), Cl^- (2p), and Cu^+ (2p) peaks. High-resolution XPS spectrum of Cu^+ exhibits two peaks at 931 and 952 eV which can be assigned to $2p_{3/2}$ and $2p_{1/2}$ of Cu^+, respectively, confirming the successful doping of Cu^+ in $Cs_2(Ag/Na)InCl_6$ lattice.

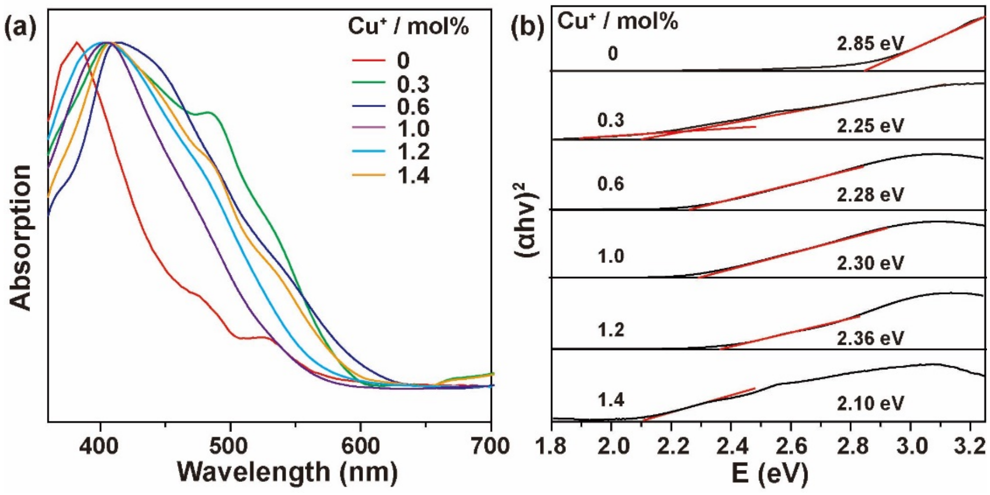

Figure S5. a) Optical absorption spectra and b) the corresponding Tauc plots of $Cs_2(Ag/Na)InCl_6$: x%Cu^+ with different Cu^+ concentrations. The crystals display a broad absorption band ranging from 350 nm to 650 nm, with its maximum shifting from 380 nm to 410 nm upon Cu^+ doping. This indicates that Cu^+ doping may lower the bandgap energy of $Cs_2(Ag/Na)InCl_6$ crystals, as confirmed by Tauc plots of the absorption spectra, whereby the optical bandgap of the crystals was estimated to decrease from 2.85 eV to 2.10 eV with increasing the Cu^+ doping concentration from 0 to 1.4 mol%.

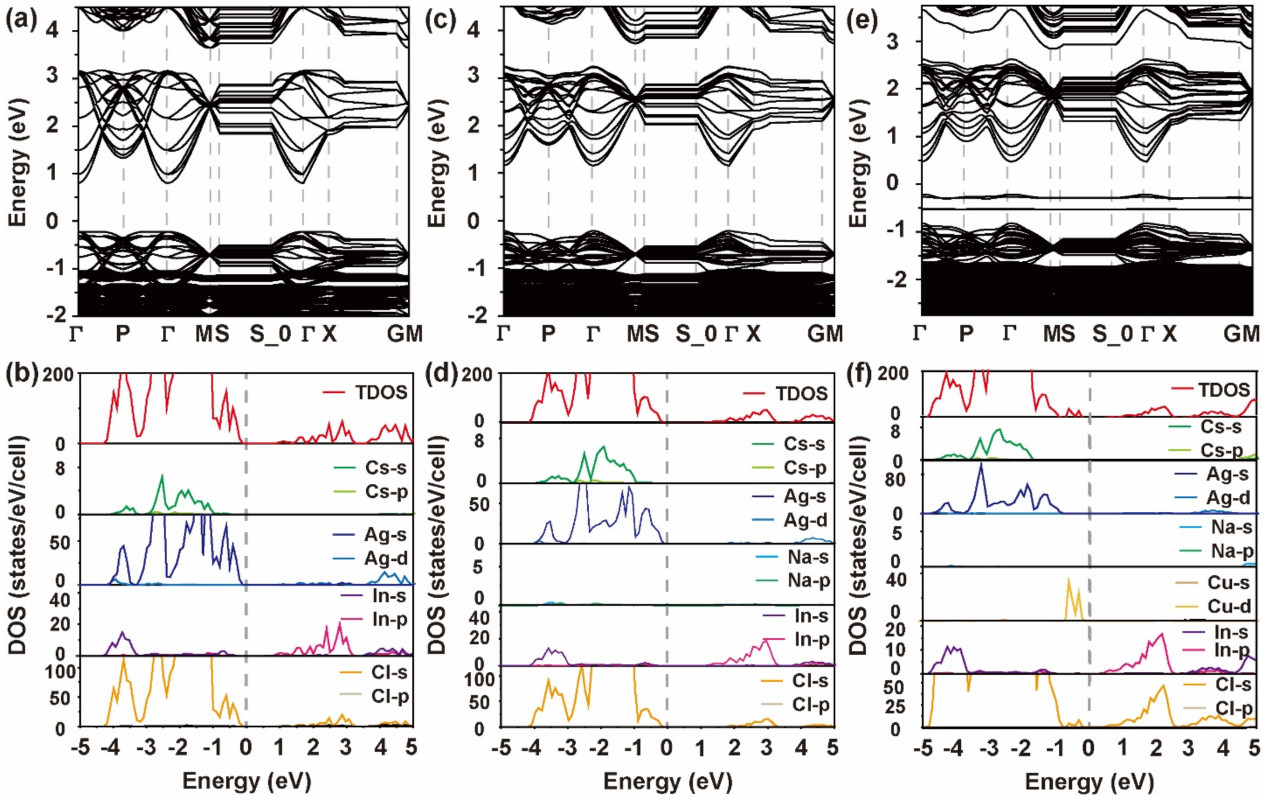

Figure S6. Band structures, total density of states (TDOS), and partial density of states of a, b) $Cs_2AgInCl_6$, c, d) $Cs_2(Ag/Na)InCl_6$, and e, f) $Cs_2(Ag/Na)InCl_6$: 5.0%Cu^+. The Fermi level is set as 0 eV in energy. For $Cs_2AgInCl_6$, the Cl-3p and Ag-4d orbitals contribute to valence band maximum (VBM) and In-5s orbitals mainly form the conduction band minimum (CBM), which result in a calculated bandgap of 1.02 eV. For alloyed $Cs_2(Ag/Na)InCl_6$, the introduction of Na in Ag sites enlarges the bandgap with the value of 1.37 eV, but the presence of Na has negligible effect on the CBM and VBM, and their main components are the same as those of $Cs_2AgInCl_6$. These results agree well with those reported by L. Manna and Du *et al.*[1] Upon Cu^+ doping, the extra Cu-3d orbitals overlap with a little Cl-3p orbitals, and they constitute new bands located at 0.3~0.6 eV above the original VBM of $Cs_2(Ag/Na)InCl_6$, and these new bands form new VBM for $Cs_2(Ag/Na)InCl_6$: 5.0%Cu^+ system. As a result, the calculated bandgap is reduced to 0.68 eV in $Cs_2(Ag/Na)InCl_6$: 5.0%Cu^+ system with a slight downshift (0.09 eV) of CBM. The calculated bandgaps are lower than the experimental values due to the general limitation of generalized gradient approximation (GGA) scheme in DFT calculation.[1, 2] Despite of the underestimation of the calculated bandgaps and the higher Cu^+ doping concentration (5.0 mol%) used for calculation, it can be concluded from the DFT calculation that Cu^+ doping brings about a new VBM above the original VBM, which results in an evident bandgap reduction of $Cs_2(Ag/Na)InCl_6$ upon Cu^+ doping.

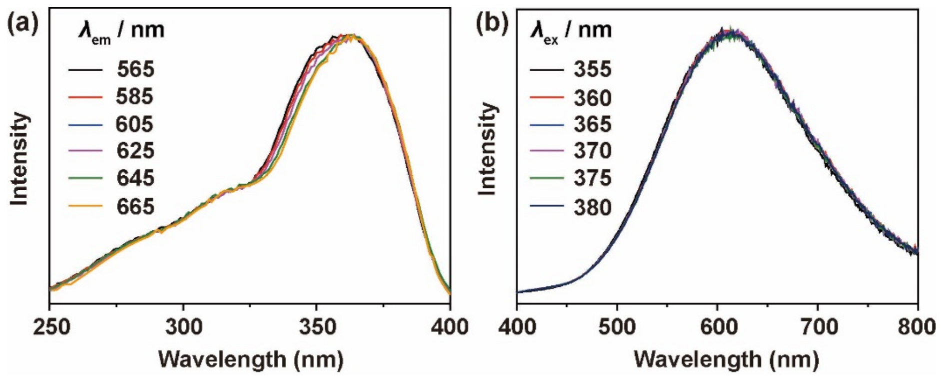

Figure S7 a) PL excitation spectra of $Cs_2(Ag/Na)InCl_6$: 1.0%Cu^+ by monitoring the STE emission at different wavelengths. b) PL emission spectra of $Cs_2(Ag/Na)InCl_6$: 1.0%Cu^+ under excitation at different wavelengths. The wavelength-dependent PL excitation and emission spectra of the crystals exhibit identical excitation and emission bands, confirming the single luminescent center of STEs in $Cs_2(Ag/Na)InCl_6$: 1.0%Cu^+ crystals.

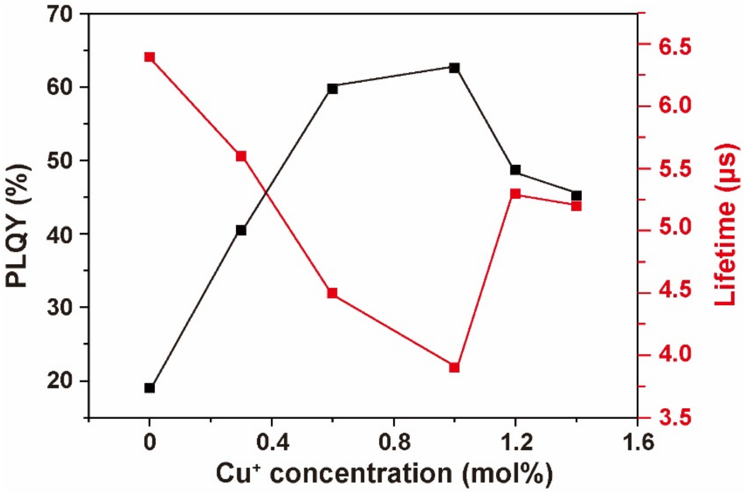

Figure S8 Comparison of the absolute PLQYs and PL lifetimes of $Cs_2(Ag/Na)InCl_6$: *x*%Cu^+ with different Cu^+ doping concentrations. The enhancement in PLQY of the crystals was accompanied with a decrease in PL lifetime.

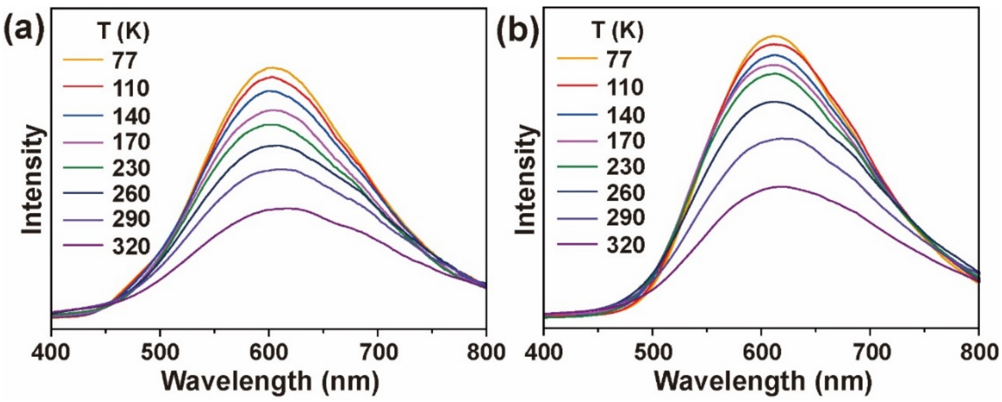

Figure S9 Temperature-dependent PL emission spectra (λ_{ex} = 365 nm) of a) $Cs_2(Ag/Na)InCl_6$ and b) $Cs_2(Ag/Na)InCl_6$: 1.0%Cu^+ in the temperature range of 77−320 K, showing decreased PL intensities of the crystals with increasing the temperature from 77 K to 320 K.

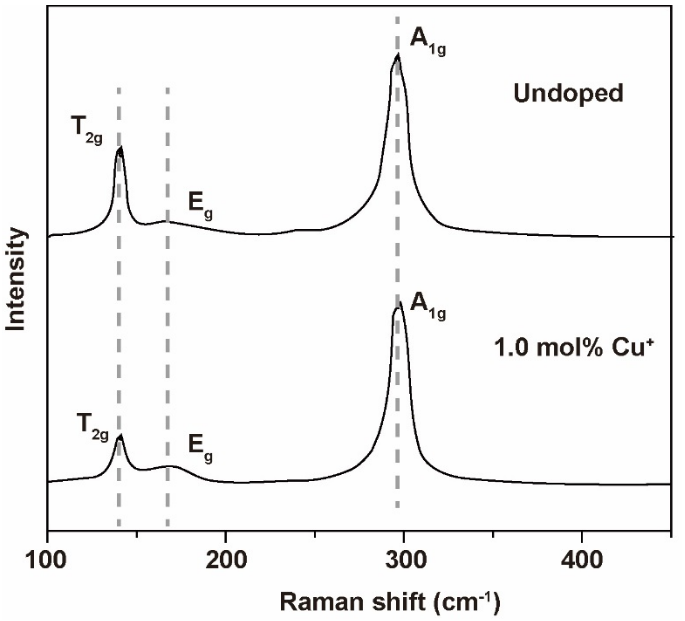

Figure S10 Raman spectra of $Cs_2(Ag/Na)InCl_6$ and $Cs_2(Ag/Na)InCl_6$: 1.0%Cu^+ under laser excitation at 532 nm. The Raman spectra of $Cs_2(Ag/Na)InCl_6$ and $Cs_2(Ag/Na)InCl_6$: 1.0%Cu^+ are dominated by the peak at 295 cm^{-1} (36.5 meV) with two less intense peaks at 170 cm^{-1} and 139 cm^{-1}. A small amount (1.0 mol%) of Cu^+ doping did not influence significantly the Raman signal. The peak at 295 cm^{-1} is ascribed to the A_{1g} longitudinal optical phonon mode associated with the symmetric stretching vibrations of Cl atoms around In atoms in the octahedron, the peak at 170 cm^{-1} is attributed to the E_g mode related to the asymmetric stretching vibrations of Cl around In, and the peak at 139 cm^{-1} is assigned to the T_{2g} mode correlated with the motion of Cs atoms.

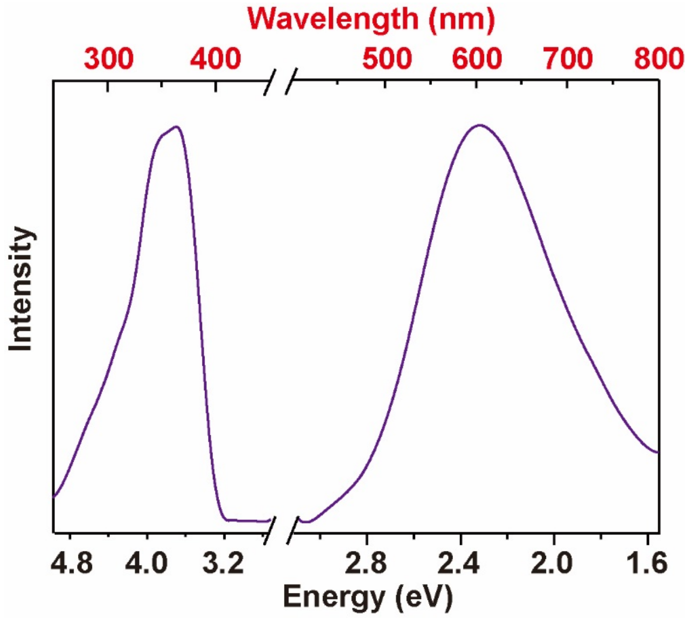

Figure S11 PL excitation (λ_{em} = 605 nm) and emission spectra (λ_{ex} = 365 nm) of $Cs_2(Ag/Na)InCl_6$: 1.0%Cu^+, showing broadband (≈590 meV) and large Stokes shift (240 nm) of the STE emission.

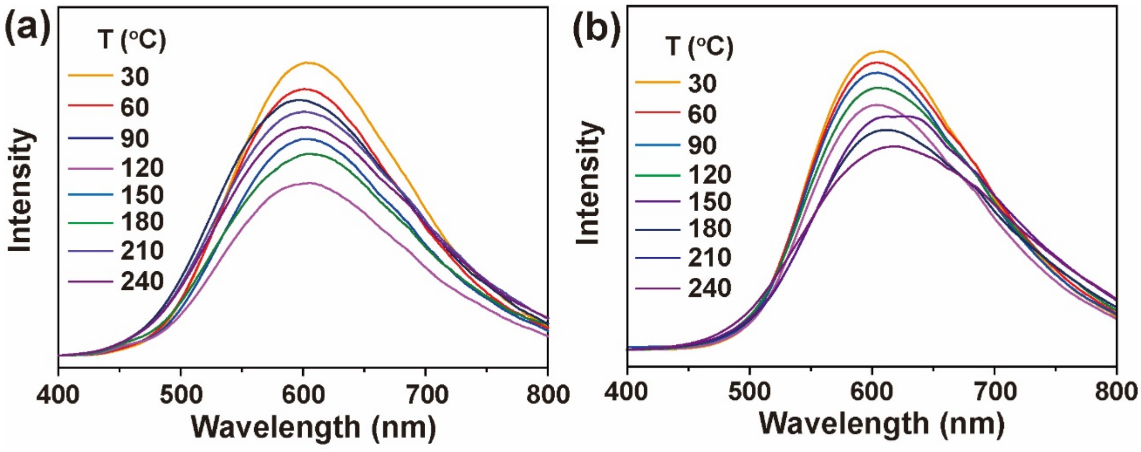

Figure S12 Temperature-dependent PL emission spectra (λ_{ex} = 365 nm) of a) $Cs_2(Ag/Na)InCl_6$ and b) $Cs_2(Ag/Na)InCl_6$: 1.0%Cu^+ in the temperature range of 30−240 °C, showing improved thermal stability of the crystals upon Cu^+ doping.

Reference

[1] a) F. Locardi, E. Sartori, J. Buha, J. Zito, M. Prato, V. Pinchetti, M. L. Zaffalon, M. Ferretti, S. Brovelli, I. Infante, L. D. Trizio, L. Manna, *ACS Energy Lett.* **2019**, *4*, 1976; b) Z. Zeng, B. Huang, X. Wang, L. Lu, Q. Lu, M. Sun, T. Wu, T. Ma, J. Xu, Y. Xu, S. Wang, Y. Du, and C.-H. Yan, *Adv. Mater.* **2020**, *32*, 2004506.

[2] Z. Xie, S. Lin, Z. Wang, *Ceram. Int.* **2018**, *44*, 15912.
